# Role of the consent form in UK veterinary practice

**DOI:** 10.1136/vr.105762

**Published:** 2020-10-15

**Authors:** Carol Gray

**Affiliations:** School of Law and Social Justice, University of Liverpool, Liverpool, Merseyside, UK

**Keywords:** informed consent, consent forms, veterinary medicine, veterinary clinics

## Abstract

**Background:**

Informed consent from the client is required before veterinary professionals may administer treatment or perform surgery on an animal patient, except in an emergency. This study investigates the potential role(s) of the consent form in the consent process in the UK.

**Methods:**

Thematic analysis was carried out on the text contained in 39 blank consent forms sourced from veterinary practices in the UK. Analysis was conducted at the levels of topical survey and thematic summary.

**Results:**

Consent forms were used to authorise procedures, to define proposed treatment, to offer or recommend additional procedures, to convey the risks of treatment and to document the client’s financial obligations. None of the forms analysed provided sufficient space to document the accompanying conversation. Notable omissions from the submitted forms included options for treatment and benefits of treatment.

**Conclusions:**

The consent form acts as a record of the procedure to be performed, the associated costs and the status of the person giving consent. However, from this analysis, it often fails to record the detail of the consent discussion, an essential part of the consent process. A proposal for an improved version of a veterinary consent form is provided.

## Introduction

Informed consent, which authorises human medical treatment, requires that the person giving consent is competent, has been given adequate information about the proposed treatment and is acting voluntarily.^1^ According to professional ethical guidance given by the Royal College of Veterinary Surgeons (RCVS),^2^ these requirements of valid consent still apply in veterinary medicine, even though consent is given by a third party on behalf of the patient. Previous authors have compared veterinary and paediatric medical consent,^3 4^ suggesting that comparisons between human and veterinary medical consent are justified.

### The consent form in human healthcare

Currently, the law governing human medical treatment in the UK recognises consent that is given (1) verbally, (2) through a person’s behaviour or (3) in writing.^5^ Although the recording of consent in writing offers evidence that a discussion has taken place, it is not proof of consent. A consent form is therefore not a legal requirement for most forms of medical treatment, merely acting as a record of the accompanying conversation.

Two major problems have been identified with consent forms used in human healthcare. The first lies with the purpose of the form, and its perceived role as ‘protecting’ the institution or healthcare professional providing the treatment. The second problem concerns the utility of the form, both as a conveyor of essential information and as a record of the conversation between healthcare provider and patient.

Perception of the form’s purpose as an instrument of harm avoidance has been confirmed by studies showing that 46 per cent of hospital patients^6^ and 60 per cent of dental patients^7^ believed that the main function of the forms was to protect the hospital or practice from litigation. Findings from the latter study are perhaps particularly relevant to veterinary practice; in both settings, patients and clients enter into a financial contract for treatment with the healthcare provider. If the main purpose of consent is to enable the patient to be fully involved in medical decision-making, then such views should raise concerns. Perhaps the language used on consent forms leads to patients’ beliefs that the process is designed to protect those who provide treatment and not those who receive it.

Turning to the utility of the form, much research in human medicine involves assessment of the printed text, using established grade level readability measures^8^ to investigate the simplicity of the language. Such an approach assumes that the main method of conveying essential information to the person giving consent is via the printed text on the form. The focus on text perhaps even reinforces the ‘harm avoidance’ approach to consent, implying that if the language used is clear and simple, the patient must have understood what he/she was signing.

However, it is the form’s role as a record of the consent conversation that is perhaps under-researched. Few authors have examined this area, although some have included the consent conversation as an adjunct to research focused on the form itself,^9^ while others have examined the respective roles of the form and the conversation with a view to improving the consent process.^10^

In the UK, the advice given to healthcare professionals is that ‘*(f)or significant procedures, it is essential for health professionals to document clearly both a patient’s agreement to the intervention and the discussions which led up to that agreement*’.^11^

### The consent form in veterinary medicine

Much of the information surrounding the use of consent forms in veterinary practice is gleaned from professional ethical guidance. For the profession in the UK, the RCVS advises that ‘…. *signed consent forms are required for all procedures including diagnostics, medical treatments, surgery, euthanasia and when an animal is admitted to the care of a veterinary surgeon’* 2 while reiterating that ‘*consent forms should be viewed as an aid to consent, in conjunction with a discussion with the client’*.^2^

However, research into veterinary consent forms is sparse. An early ‘professional advice’ paper advised the use of consent forms to protect the veterinary surgeon from allegations of trespass.^12^ This ‘harm avoidance’ role is reinforced by a study conducted at a large veterinary referral hospital, which found that one-third of respondents thought consent forms were used to protect the veterinary surgeon, and one-fifth thought their main purpose was to protect the hospital.^13^

The utility of veterinary consent forms has been investigated in the research setting, but only in the USA. Using grade-level readability measures, this study found that veterinary forms have lower readability than their human medical research equivalents.^14^ No similar studies have been conducted in the UK. If consent forms for veterinary treatment also use incomprehensible language, then their role as providers of information may be undermined.

The RCVS refers to consent as ‘*an essential part of any contract*’, regarding financial estimates as part of the consent conversation and recommending that these ‘*should also be documented on the consent form, or on an attached detailed estimate’.* 2 The veterinary consent form therefore has a dual role, also acting as a record of the contract for payment of veterinary services. The need for clear and understandable information applies both to contract, where terms need to be clearly defined, and to consent.

## Methods of data collection

The role of the consent form in UK veterinary practice was investigated using qualitative analysis to construct its role in the consent process. Consent forms were obtained from a selection of practices via requests placed on social media, from direct email contact with veterinary practices and from personal contacts of the author. The request sought blank consent forms, thus removing data protection issues arising from handling client details.

Sixty forms were submitted. From these original forms, a total of 39 were included in the analysis. Excluded forms were duplicates, sought consent for other procedures (such as euthanasia) or were submitted from outside the UK. All forms had practice details removed before being uploaded to qualitative analysis software, QSR NVivo, for organisation and storage before initial coding and thematic analysis. The author was responsible for anonymising the forms and for performing the analysis, as part of a programme of research for a doctoral thesis. The forms were not assessed for their readability scores.

## Data analysis

Evaluation of the forms was performed using theoretical thematic analysis.^15^ An open approach to coding, with criteria defined through coding notes (for an example of these, see table 1) and using constant comparison to check for validity, resulted in mainly semantic (data-derived) themes, with some latent themes derived from the theoretical framework of professional ethical guidance.^16^

**Table 1:** Examples from coding book

Code	Use for	Do not use for	Examples
Risk disclosure: generic	Reference to generic risks of anaesthesia and/or surgery	Reference to specific risk or magnitude of risk involved	*‘there is a risk with every anaesthetic’*
Risk disclosure: specific	Reference to specific risk or magnitude of risk	General reference to non-specific risks	*“I accept that possible complications from the procedure may occur such as sepsis, wound breakdown, haemorrhage and anaesthetic reaction”*

Analysis was conducted following the levels of analysis proposed by Sandelowski and Barroso.^17^ An initial topical survey remained close to the original wording on the forms analysed. Combination of themes resulted in a thematic summary. Section A presents the topical survey, while Section B draws on the thematic summary (table 2). Forms are numbered as ‘CFxx’, where CF is consent form, followed by a randomly assigned number.

**Table 2:** Levels of analysis for data from consent forms, from Sandelowski and Barroso^17^

Topical survey (initial codes)	Thematic summary
Description of procedure(s) Offering additional procedures Recommending additional procedures	The role of consent forms in describing the proposed procedure, offering other procedures and documenting options for treatment.
Eliciting health details Outlining risks of GA Outlining risks of surgery Listing postoperative complications Listing requirements for aftercare Referring to uncertainty of outcome	The role of the form in conveying the risks and benefits of proposed treatment.
Estimating costs Contracting for payment Charging for additional services Referring to payment for unexpected outcomes	The role of the form in evidencing a financial contract.
Confirming ownership or authorised agency Confirming consent Seeking consent for unspecified procedures/unlicensed drugs Confirming understanding	The role of the form in authorising treatment.

### Section A: topical survey

RCVS professional ethical guidance recommends confirming that the correct person is giving consent, that this person has been given the information about the proposed treatment, options, risks and benefits, understands the information and can indicate consent, usually in writing.^2^

#### Describing the procedure and offering others

All consent forms analysed provided space for a description of the procedure being undertaken. The prompt for identifying the procedure varied amongst forms. For example, ‘*Operation/procedure:* ________________’ or ‘*Surgical procedure:* _________’ appeared on 20 forms; ‘*Proposed operation:* _____’ on 3 forms, and ‘*Reason for admission:* _________’ on 4 forms.

However, little space was provided for giving more information about the surgical procedure, suggesting that to fulfil the requirements for informed consent, more detail would need to be given during the accompanying discussion. There were no examples among the forms studied where space was allocated for recording options for treatment.

The ‘additional procedures’ theme included both recommending and offering additional procedures that could be performed at the same time as the identified surgical procedure. The division into two subthemes was decided primarily on the language used on the forms, suggesting which of the parties involved made the decision for the optional procedure. ‘Offering’ involved listing the available additional procedure(s) without a strong recommendation from the veterinary practice, thus apparently leaving the decision to the client. ‘Recommending’ involved a strong written recommendation for a specific procedure, although this was often accompanied by the ability to ‘opt out’.

#### Offering additional procedures

Additional services offered included the provision of postoperative recovery diets or the option of having laboratory investigations performed on any lumps removed. Many forms offered preoperative blood tests to all clients whose animals were scheduled for surgery, with some including the financial implications for the client on the form.

In the examples shown in table 3, the wording suggests that clients were given options and made the decisions. It is not known to what extent the client would be helped by the person obtaining consent. The options involved additional costs, which were sometimes explicit and sometimes hidden, for example, as a ‘small additional fee’. In some cases, therefore, clients were given the option of additional services without information about the costs involved.

**Table 3:** Offering additional procedures on consent forms

Sample text	Source
*“Would you like your pet to go home with a special postoperative diet pack upon discharge? (There will be a small additional fee for this) YES/NO”*	CF33
*“In case of mass removal, do you wish to have histopathology Yes/No”*	CF9
“*Would you like your pet to have a pre-anaesthetic blood test? YES/NO*”	CF2
*“Would you like a blood test before your pet’s anaesthetic? (£41.34)?”*	CF58

#### Recommending additional procedures

On some forms, the veterinary practice either recommended procedures, or included a statement that the practice may carry out certain procedures and charge the client accordingly, for example, in treating any parasites that were found on the patient, or in using laser therapy to accelerate wound healing. Several practices made strong recommendations for preoperative blood tests, either for every patient or only for certain patients, however the client was usually able to opt-out.

The examples shown in table 4 strongly directed clients to accept certain additional procedures or actions. however, as with ‘offered’ procedures, the costs were sometimes hidden.

**Table 4:** Recommending additional procedures on consent forms

Sample text	Source
*‘Please note that appropriate flea control will be applied where necessary at the owners* (sic) *expense’.*	CF23
*“At the time of operation and post op check we automatically perform subject to availability laser surgery of the wound to speed up the healing process at a cost of £10.00. Would you like to opt out? Yes 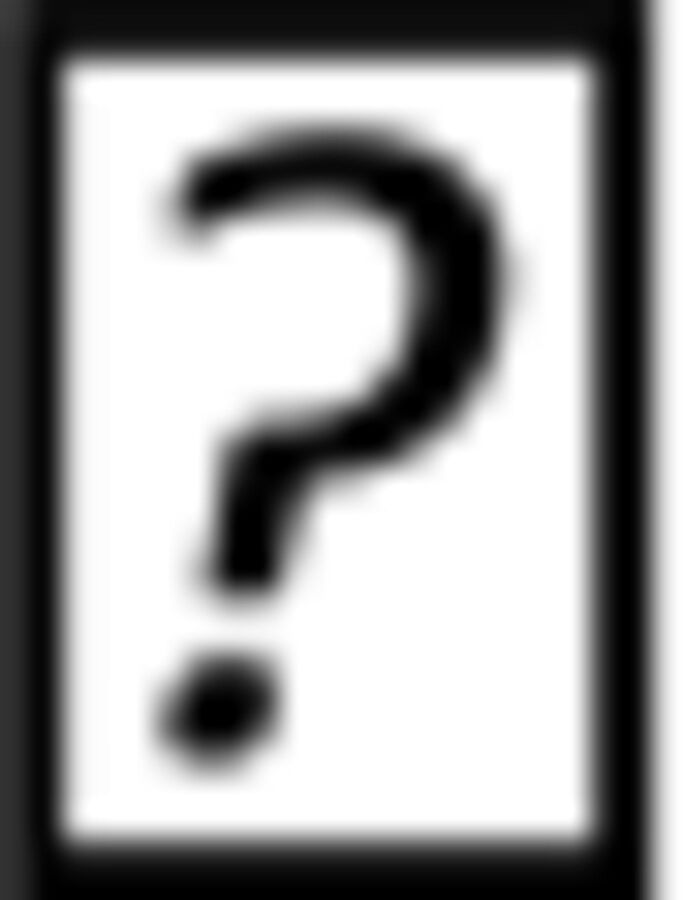 No 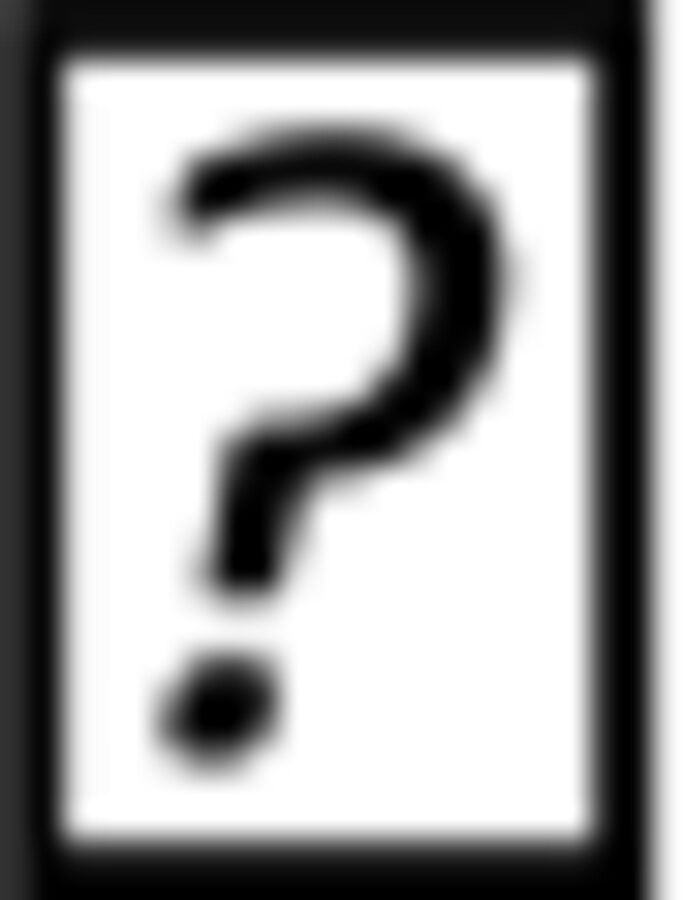 ”.*	CF50
*“We recommend a pre-anaesthetic blood test to eliminate many pre-existing problems that may not be evident physically, but could lead to complications”.*	CF1
*“We will perform a pre-anaesthetic blood test if we believe it to be necessary—this is usually in ill or elderly animals. This does involve an extra cost. If you do not want us to do this test please tick the box”.*	CF33

#### Outlining risks of anaesthesia and surgery, and uncertainty of outcome

The majority of the forms analysed mentioned risks. For elective surgery, the main risk that needs to be conveyed is the statistically small risk of death occurring under general anaesthesia.^18^ On the forms analysed, the risks involved in sedation and/or general anaesthesia were often described in generic terms, but required the client to confirm that they understood the risks. Sometimes the risks were clarified in terms of the status of the patient, but in the first two examples shown in table 5, the nature of the risk is not explained. It may be that the nature of the risks involved will be covered during the accompanying discussions, or it may be that clients will be left to construct their own ideas of the nature of the risks involved.

**Table 5:** How risk and uncertainty are conveyed via consent forms

Sample text	Source
*“I understand that all anaesthetic and surgical procedures involve some risk to the animal”.*	CF1
*“Operations and procedures, however small, which require sedation or anaesthesia to facilitate their performance, carry a slight risk to the patient. These risks may be increased if your pet is old, overweight or ill and in a number of other circumstances”.*	CF6
*“I acknowledge and agree to the risks involved and understand that in extreme circumstances these may include loss of life”.*	CF44
*‘Risks and complications associated with diagnosis and treatment may include: ____________’*	CF32
*“I accept that possible complications from the procedure may occur such as sepsis, wound breakdown, haemorrhage and anaesthetic reaction. Further possible complications: ________________”*	CF50
*“I also accept that the success of medical or surgical treatment cannot be guaranteed”.*	CF21

Several forms explained the risks in more detail, although they stopped short of explicitly stating that the serious risk is death (table 5). One form left space for documenting the risks discussed during the consent discussion, thus allowing integration of the consent conversation and the form. Similarly, another listed common adverse outcomes following surgery, leaving space to document additional risks that had been discussed.

None of the forms analysed left space for listing the benefits of the proposed surgery, nor for recording any discussion about these benefits, which is perhaps particularly relevant to decisions about neutering surgery when the surgery is not essential for the animal’s health.

A few forms referred to the uncertainty of the results of the treatment, thereby reinforcing the lack of guaranteed outcome that applies to medical procedures (table 5). In veterinary medicine, the involvement of financial obligation on the part of the client has similarities with a written contract. However, even when medical treatment involves a contract between service provider and patient, it would be unusual to guarantee success,^19^ so it is debatable whether such a statement is required on consent forms. Nevertheless, one form included a statement that the client would still be required to pay in the event of an adverse outcome.

#### Estimate of costs, charges for additional services or unexpected outcomes

Most of the forms analysed provided space for estimated costs of treatment on the consent form. For the others, it was unclear whether there was a separate written estimate provided, or if the discussion was documented in the patient’s clinical records. Some of the language used on the forms pertained to the financial contract, an aspect that was sometimes reinforced by a request for details of the proposed payment method at the time of consent (table 6).

**Table 6:** The form as a contract

Sample text	Source
*“I understand that the complete fee is due for payment when I take my pet home”.*	CF1
*“I understand that all fees must be settled at the end of surgery. I will pay my account by □Cash □Cheque □Card”.*	CF12
*‘Complications are rare but can occur……. (…………….) …… if they do occur then consultations within the first 2 weeks are included in the price of the operation. All costs of medication needed during this time, further consultations beyond 2 weeks or any repeat surgery if indicated will be additional to the costs involved initially’.*	CF60

One form clearly outlined the position regarding payment for postoperative complications, specifically the relative obligations of the practice and the client, thus clarifying the practice’s contract terms.

#### Confirming suitability to consent

According to RCVS professional ethical guidance, the client ‘*may be the owner of the animal, someone acting with the authority of the owner, or someone with statutory or other appropriate authority. Care should be taken when the owner is not the client’*.^2^

All forms analysed had provision for recording the identity and contact details of the client and the animal patient.

#### Ownership

Most forms provided the option for the client to sign as the owner or the owner’s agent, but only required ticking of the relevant box, or deletion of whichever term was not appropriate.

Some forms, however, included a statement requiring the client to confirm that they were the owner of the patient, or that they had the owner’s permission to make treatment decisions (table 7). Several forms included confirmation that the person signing the form was at least 18 years of age, as required of both parties to a contract in England. None of the forms analysed required confirmation of age by the person taking consent, but in view of the possibility of student veterinary nurses (who may be 17) obtaining consent in some circumstances, it is probably wise that both parties confirm that they meet this age requirement on the form.

**Table 7:** Confirming authority to consent

Sample text	Source
*“I am the owner or I am acting with the full knowledge and authority of the owner”.*	CF29
*“I am the owner or agent of the above animal and have the authority to give this consent. I am over 18 years of age”.*	CF23
*“I have read and understood this form and hereby voluntarily give my consent”.*	CF12
*“I understand that it may be necessary to use an unlicenced* (sic) *drug during the above procedure. I do/do not give my consent”.*	CF15
*“I understand that there may be occasions when it will be necessary to use medicines which, while not specifically authorized for the treatment of this species, may be used legally when justified clinically. I have been made aware, and accept, that there may be unknown side-effects associated with the use of such medicines in this species, and I consent to their use”.*	CF42
*“I hereby give consent to and authorise the performance of such procedures as are necessary and desirable in the exercise of the veterinary surgeon's professional judgement”.*	CF23
*“…. if I can't be contacted the Veterinary Surgeon will act in the best interests of my animal. I accept this may incur additional costs”.*	CF29
*“Declaration by Veterinary Surgeon: I confirm I have explained the risks of the anaesthetic and procedure in terms that I judged were understood by the owner/authorised agent”.*	CF31

#### Confirmation of consent

All forms analysed had a form of words indicating that specific consent was given, via a clear statement of intent, for example, “*I hereby give permission* ….” (most commonly), “*I give my consent to* …” (eight forms); “*I authorise”* (three forms).

On some forms, there was also an opportunity for the client to confirm understanding, and lack of coercion (table 7).

#### Consent for the use of unlicensed products

The RCVS requires written consent for the use of unlicensed medicines.^20^ This was usually achieved through a generic statement, although one form provided comprehensive information about the use of unlicensed drugs (table 7).

#### Proceeding without consent

Several forms sought consent for unforeseen treatment that may have been required during the procedure. Sometimes this was expressed as a comprehensive statement suggesting that the veterinary surgeon could perform any procedure deemed necessary, with only some forms clarifying the financial aspect of this ‘blanket’ consent (table 7). The most common format of this statement was ‘*together with any other procedures which may prove necessary’,* which appeared on approximately half of the forms analysed; this wording is taken directly from the RCVS’s specimen consent form.^21^

#### The role of the veterinary professional in the process

Some forms required the veterinary professional involved in the process to confirm that the client had been given the information in a suitable format (table 7), and several forms required a counter-signature by the person obtaining consent, although only one form provided a ‘tick-box’ to allow confirmation that a copy had been provided to the client.

### Section B: thematic summary

Although a consent form is neither a legal requirement, nor proof of consent, a well-designed consent form can provide a substantial foundation for physician-patient discussions, despite the conclusion from a large-scale American study of hospital consent forms that ‘*forms as designed have limited value*”^22^

Conceptual analysis of the submitted veterinary consent forms considered the extent to which these forms could play a role in obtaining informed consent, by invoking the RCVS requirements for consent (ie, the nature, purpose and benefits of proposed treatment, common and serious risks, financial estimates and options for treatment)^2^ and examining how well the forms could provide evidence of discussion of these criteria.

#### The role of the form in defining the proposed procedure, offering other procedures and offering alternatives

Few of the forms analysed provided enough space to describe the proposed procedure in sufficient detail to satisfy the requirements of informed consent, meaning that this aspect would need to be covered more comprehensively in the accompanying discussion. Many forms did contain the offer of additional procedures, most commonly preoperative blood tests. Some of the forms analysed resembled a menu of additional purchases, raising questions about how much information and how much help the client is given when making decisions about these additional items.

None of the forms analysed provided space to document alternative treatments. As the RCVS regard ‘options for treatment’ as an essential component of informed consent, this would also need to be covered during the consent discussion.

#### The role of the form in conveying the risks and benefits of proposed treatment

When communicating risks, the forms analysed were often non-specific about (a) the type of risk and (b) the level of risk. Nevertheless, at least there was some reference to the risks involved, whereas none of the forms included any reference to the benefits of treatment. This topic could be covered in the accompanying discussion. However, if, as is recommended,^2^ the client is given a copy of the form to take away in advance of the proposed procedure, then the balance between risks and benefits needs to be clearly conveyed to facilitate the client’s deliberation.

#### The role of the form in evidencing a financial contract

As most veterinary treatment incurs costs, the consent form also acts as a record of the contract for payment of veterinary services. This dual purpose is not without problems. In veterinary healthcare, like in human healthcare in many other countries, consideration, or payment, for healthcare is inextricably linked to consent. This means that the consent form represents a written contract. However, like any other contract, it could be voided if the terms are too vague. As Bix observes, *‘(o)ne cannot consent to terms …. (……)…… without knowledge of the terms’*.^23^

With respect to clear contract terms, the most obvious deficiency of some of the forms analysed was the failure to state estimated costs clearly. Some forms included the provision for the veterinary surgeon to carry out unspecified procedures, based on what they considered necessary. These forms suggested that for unforeseen procedures, when the owner was not contactable, decisions would be made in the ‘best interests’ of the animal, but the owner would be charged for the additional procedures. The inclusion of statements referring to the veterinary surgeon’s ability to carry out ‘any treatment deemed necessary’ may diminish the form’s role as a written contract. To achieve the latter purpose, the form would need to include the proposed treatment and any other treatments that may be necessary, with estimated costs for each.

#### The role of the form in authorising treatment

All forms analysed used ‘quasi-legal’ terminology, which may originate from the RCVS’s own sample consent form.^21^ There are drawbacks to this ‘legal’ appearance. Clients may not read the form carefully, assuming that they must sign the form to allow the procedure to go ahead, or they may be either falsely reassured or confused by the legal terminology.^24^ The legalistic appearance and language may contribute to clients’ belief that the form is designed to protect the veterinary practice.^13^

At some point in every form analysed, the client was asked to formally give consent to the proposed treatment, an action that also required confirmation of their suitability to consent, as the owner (or owner’s agent) of the animal patient.

A few forms required input from the person obtaining consent, through a signature and/or confirmation that information had been given to the client in a way that maximised understanding. In the author’s opinion, the role of the form as a ‘quasi-legal’ record of a consent discussion necessitates both parties recording their part in the process.

#### Limitations of study

This study was conducted in a single jurisdiction, with a limited number of forms. However, the consistency of the themes that were developed suggested that no ‘new’ areas of the consent form would have been uncovered by increasing the number of forms analysed. The analysis was conducted by a single researcher, as part of a larger study. Although involving a second person to deliberate and decide on themes would have increased validity, the proximity of themes to the language used on the original forms perhaps lessens this requirement.

## Discussion

This study of veterinary consent forms is the first to analyse the language used on these forms in the veterinary medical setting. It is also the first study to confirm that consent forms fulfil multiple roles in veterinary practice. The perceived view of the form as a way of protecting the veterinary practice against litigation by clients is reinforced by the use of ‘quasi-legal’ language in many of the forms analysed. Translating the text into everyday terms could increase the utility of the forms for both parties.

The consent form has other roles that have become apparent through this analysis. It can act as a ‘shopping list’ to offer the client a range of additional procedures and extras, expanding on its role as a commercial document. It can serve as an aide-memoire for the person taking consent, by listing topics that should be covered and thereby providing structure to the discussion. Perhaps most importantly, it can provide written evidence that there has been a discussion about the proposed treatment, and that the client has authorised and agreed to pay for a specific procedure.

To provide evidence of the consent discussion, the form needs to include sufficient blank space to record the main points of the conversation. The forms analysed rarely provided sufficient space to record a consent discussion, with most of the layout occupied by text. A consistent finding was the length of the forms submitted, none extending to more than two sides of A4 paper, with the text squashed into the available space. Such restriction makes it difficult for the form to provide useful evidence of the wider aspects of the discussion, such as details of alternative treatments, and the risks and benefits of the proposed treatment.

In an effort to translate these findings into a practical format, the author has designed a new model consent form, available in online supplementary appendix A. Based on the layout of the RCVS Specimen Form of Consent for Anaesthesia and Surgery,^21^ it incorporates some ideas from the forms analysed for this study, and some ideas from consent forms used in human medicine. Although presented as a suggested ‘improved model’, incorporating the findings of this study and current RCVS guidance on consent, it has not yet been evaluated in practice.

10.1136/vr.105762.supp1 Supplementary data

Its main revisions are the use of more ‘lay’ language, the provision of more space for documenting the consent conversation, removal of the phrase implying that additional procedures can be performed without specific client consent and provision for the signatures of both parties.

Finally, it is worth reiterating that consent should be viewed as ‘a series of conversations’,^10^ with the revised form proposed as a method of providing a more complete record of these conversations.
