# Supplementary material for: Role of the consent form in UK veterinary practice
Source: Vet Rec. 2020 Oct 15;187(8):318. doi: 10.1136/vr.105762 (PMC7606496; doi:10.1136/vr.105762)
Supplement: Supplementary data [file vetrec-2019-105762supp001.pdf]

SPECIMEN FORM OF CONSENT FOR ANAESTHESIA, CLINICAL AND SURGICAL PROCEDURES, BASED ON RCVS VERSION

Owner details

Printed label from client records

Patient details

Printed from patient records

To be completed by person taking consent:

Details of the **Operation/Procedure** ____

Alternatives discussed ____

Risks discussed ____

____

Benefits to the patient of having this procedure

____

Pre-operative blood tests recommended for this patient, **YES/NO:** cost explained ☐

Client agrees to/requests blood tests ☐ Client declines blood tests ☐

Any other procedures requested by client, with costs

____

The **cost** of the procedures described above (tick as appropriate)

☐ will be: £______________ OR

☐ will be within the range: £____________ to £_________

Inclusive of: VAT ________

Any financial limit placed by owner? **YES/NO** Amount:

To be completed by owner or authorised agent:

In order to protect the welfare of my animal, in the unlikely event of an emergency, or where additional pain relief or sedation may be required, I understand the veterinary surgeon may decide to use medicines that are not authorised for use in [state species]. ☐

Contact number in case of emergency:______________________________________

☐ I accept this estimate of costs and agree to pay on collection of the animal.

☐ I agree that the proposed procedures have been explained to me, I have had the opportunity to ask questions, and I understand and accept the risks involved.

☐ I give my consent to the treatment agreed

☐ I am the owner of this animal **OR**

☐ I am not the owner, but I have the authority to act on behalf of the owner of the animal described above

☐ I confirm that I am over the age of 18

Signature of person giving consent:

____

Date:

I confirm that I am over the age of 18, and that I have explained the proposed procedure, alternatives, risks and benefits

Signature of person obtaining consent:

____

Date:

Copy given to client: ☐
